# Implementation of evidence on management of pleural diseases: insights from a territory-wide survey of clinicians in Hong Kong

**DOI:** 10.1186/s12890-022-02196-4

**Published:** 2022-10-24

**Authors:** Macy M. S. Lui, Yiu-Cheong Yeung, Jenny C. L. Ngai, Kit-Man Sin, Yi-Tat Lo, Alice P. S. Cheung, Ka-Yan Chiang, Yu-Hong Chan, Ken K. P. Chan, Connie H. K. Lam, Wei-Lam Law, Siu-Leung Fung, Wai-Kei Lam, David C. L. Lam, Lam-Hin Shek, Ida W. Y. Wong, Anthony P. Y. Yau, Yun-Chor Gary Lee, Johnny W. M. Chan

**Affiliations:** 1 Department of Medicine, Queen Mary Hospital, The University of Hong Kong, Hong Kong Island, Hong Kong; 2 Department of Medicine & Geriatrics, Princess Margaret Hospital, Kowloon, Hong Kong; 3 Department of Medicine and Therapeutics, Prince of Wales Hospital, The Chinese University of Hong Kong, Shatin, Hong Kong; 4 Department of Medicine, Tuen Mun Hospital, Tuen Mun, Hong Kong; 5 Department of Medicine, Pamela Youde Nethersole Eastern Hospital, Chai Wan, Hong Kong; 6 Department of Medicine & Geriatrics, United Christian Hospital, Kwun Tong, Hong Kong; 7 Department of Medicine, Queen Elizabeth Hospital, Kowloon, Hong Kong; 8 Tuberculosis & Chest Unit, Grantham Hospital, Aberdeen, Hong Kong; 9 Department of Medicine, North District Hospital, Sheung Shui, Hong Kong; 10 Department of Medicine & Geriatrics, Caritas Medical Centre, Sham Shui Po, Hong Kong; 11 Department of Medicine, Haven of Hope Hospital, Tseung Kwan O, Hong Kong; 12 Department of Respiratory Medicine, Kowloon Hospital, Kowloon, Hong Kong; 13 Department of Respiratory Medicine, Sir Charles Gairdner Hospital, Perth, Australia; 14 Institute for Respiratory Health, School of Medicine, University of Western Australia, Perth, Australia

**Keywords:** Pleural diseases, Malignant pleural effusion, Exudative pleural effusion, Pleural infection, Pneumothorax, Ultrasonography, Training, Survey

## Abstract

**Background:**

Major advances in management of common pleural diseases have taken place in the past decade. However, pleural diseases are often managed by physicians of diverse training background and research on implementation of new knowledge is scanty. We aim to evaluate the practice pattern in pleural medicine among physicians in Hong Kong, for identification of possible gaps for clinical service improvement.

**Methods:**

The Hong Kong Thoracic Society undertook a cross-sectional questionnaire survey in 2019, targeting clinicians of various subspecialties in internal medicine and levels of experience (basic and higher trainees, specialists) from twelve regional hospitals of diverse service scopes throughout Hong Kong. Respondents were selected by non-probability quota sampling. The questionnaire tool consisted of 46 questions covering diagnostic and therapeutic aspects of common pleural diseases. The responses were anonymous, and analysed independently using SPSS statistics software.

**Results:**

The survey collected 129 responses, 47(36%) were from clinicians specialized in respiratory medicine. Majority of the respondents (98%) managed pleural diseases, including performing pleural procedures in their practice. Fifty-five percent of all the respondents had not received any formal training in transthoracic ultrasonography. A significant proportion of clinicians were unaware of pleuroscopy for investigation of exudative pleural effusion, indwelling pleural catheter for recurrent malignant pleural effusion, and combined intra-pleural Alteplase plus DNase for treatment of pleural infection (30%, 15% and 70% of non-respiratory clinicians respectively). Significant heterogeneity was found in the management of pleural infection, malignant pleural effusion and pneumothorax among respiratory versus non-respiratory clinicians. Contributing factors to the observed heterogeneity included lack of awareness or training, limited accessibility of drugs, devices, or dedicated service support.

**Conclusion:**

Significant heterogeneity in management of pleural diseases was observed among medical clinicians in Hong Kong. Continuous medical education and training provision for both specialists and non-specialists has to be strengthened to enhance the implementation of advances, improve quality and equity of healthcare provision in pleural medicine.

**Supplementary Information:**

The online version contains supplementary material available at 10.1186/s12890-022-02196-4.

## Introduction

Pleural diseases are common medical conditions in Hong Kong and Mainland China, and management of pleural diseases often requires admission to the hospitals [1]. According to data derived from the electronic health database (Clinical Data Analysis and Reporting System, CDARS) of the Hospital Authority Hong Kong, admission episodes principally related to pleural effusions to various medical departments saw a 70% increase from 2009 to 2019. Increasing burden of chronic heart, renal and liver diseases associated with aging population contributes to the rising incidence of pleural effusions. Malignant or para-malignant pleural effusions are common complications along the course of all sorts of cancers, which lead to repeated hospitalization and healthcare costs as reported in recent epidemiological studies [2, 3]. More awareness and knowledge about pleural infection has revealed it as a common medical problem worldwide with detrimental implications on morbidities, mortality and healthcare burden [4]. In face of the rising service demand, it is paramount for the healthcare system to implement evidence and keep up with the practice that can assure effectiveness, improve efficiency and safety of managing pleural diseases.

In the past decade, the field of pleural medicine has witnessed significant advances [5], with landmark publications of practice-changing multi-centred randomized controlled trials and release of clinical guidelines. In particular, there are major breakthroughs in the understanding, investigation and treatments of common pleural diseases [6], which can reduce morbidity and improve quality of care for patients. However, the incorporation of major advances into clinical practice can vary tremendously across different healthcare systems [7, 8], and there are a number of possible barriers contributing to the insufficient update and lack of implementation of guidelines and advances [9].

In the Hong Kong hospital system, doctors from general medical teams are responsible for managing patients with pleural diseases, at least in the initial phase. The knowledge and practice of internists can pose significant impact on the quality and safety of healthcare for patients with pleural diseases in Hong Kong. As such, we aimed to conduct a cross-sectional questionnaire survey among medical clinicians in Hong Kong, to understand the local practice pattern in management of common pleural diseases, the utilization of newer medications, devices and technology, and possible reasons contributing to the lack of uptake of medical advances.

## Methodology

The questionnaire survey was conducted in 2018-19 among medical clinicians working in regional hospitals in Hong Kong. Twelve hospitals were selected to represent the seven regional clusters, scopes of services (acute or rehabilitation) and support (with or without on-site cardiothoracic surgical support). Depending on the population size of the region, one to two acute hospitals with emergency departments were selected to represent each of the seven regional clusters under the Hospital Authority Hong Kong. The three hospitals specialized in chest rehabilitation were included. The twelve participating hospitals included acute hospitals with on-site cardiothoracic surgery service (Queen Mary Hospital, Prince of Wales Hospital, Queen Elizabeth Hospital, Tuen Mun Hospital), acute hospitals without on-site cardiothoracic surgery service (Prince Margaret Hospital, Pamela Youde Nethersole Eastern Hospital, United Christian Hospital, Northern District Hospital, Caritas Medical Centre), and Chest/Rehabilitation hospitals without on-site cardiothoracic surgery service (Grantham Hospital, Kowloon Hospital, Haven of Hope Hospital).

The survey targeted clinicians at all experience levels working in internal medicine. A coordinator from each hospital was responsible for sampling survey targets in their respective hospital, and distributing the questionnaire tool in printed forms (Supplementary material). The survey questionnaire consisted of forty six questions covering diagnostic and therapeutic domains of common pleural diseases. The study adopted non-probability quota sampling method, by which samples are included by judgement on a non-random basis according to relevant control characteristics (institutions, subspecialties and level of experience), so that the selected sample group is representative of the composition of the target population. The survey questionnaires were distributed to the clinicians who had consented to participate in the survey. The coordinator was instructed to include an even distribution of clinicians working in respiratory medicine and non-respiratory medicine, and of different levels of experience: specialists, higher physician trainees who had completed three-year basic training, or basic physician trainees who were within the initial three years of training). Each coordinator aimed to collect about 10 anonymous responses with no personal identifiers in envelopes within a day after the distribution. All were subsequently sent to Queen Mary Hospital for data entry and analyses.

The survey study has been approved by the Institutional Review Boards of The University of Hong Kong/Hospital Authority Hong Kong West Cluster, the Cluster Research Ethics Committees (CREC) of Joint Chinese University of Hong Kong-New Territories East, Hong Kong East Cluster, Kowloon Central/Kowloon East Cluster, Kowloon West Cluster and New Territories West Cluster. The study and all methods were carried out in accordance with the Declaration of Helsinki and relevant regulations. Informed consent was obtained from all the study participants. The manuscript followed the STROBE reporting guideline.

### Statistical analyses

As of the year 2016, there were about six hundred internists working in the twelve hospitals. Assuming two-thirds of the internists were actively involved in frontline clinical practice in general internal medicine, 120 completed responses will achieve 95% confidence interval with 7.5% margin of error in a population size of 400, using the online sample size calculator designated for survey studies (Survey Monkey, San Mateo, CA, USA) [10]. SPSS version 25 (IBM SPSS Statistics for Windows, Version 25.0. Armonk, NY: IBM Corp.) was used for statistical analysis. Pearson’s Chi square or Fisher’s exact tests were used to compare between two groups, and compare expected and observed frequencies in categorical variables. Statistical significance was defined by *p*<0.05.

## Results

One hundred twenty-nine responses were collected, with 47 (36%) respondents worked in the subspecialty of respiratory medicine while 82 respondents were from non-respiratory streams. Thirty five (27%) were basic physician trainees, 37 (29%) and 57 (44%) were higher physician trainees and specialists respectively (Table 1). Forty (31%) had more than ten years of experience in practising internal medicine. A comparison of the responses among non-respiratory and respiratory clinicians to highlighted questions was summarised in Table 2.

- *Training and utilization of transthoracic ultrasonography*

**Table 1:** Demographics of survey participants

	Total number of survey participants (*n*=129)	
	Non-respiratory clinicians (*n*=82)	Respiratory clinicians (*n*=47)
Basic physician training^a^	35	0
Higher physician training	20	17
Specialists	10	8
Specialists with supervisory role	17	22

^a^Basic physician training consists of 3-year training in general internal medicine with interim examination, which is a mandatory requirement for entry into higher training of respiratory or non-respiratory streams

**Table 2:** A comparison of the responses to questions among non-respiratory and respiratory clinicians

	Total number of survey participants (*n*=129)		*P* values
	Non-respiratory (*n*=82)	Respiratory (*n*=47)	
Completion of formal TUS training	11 (14%)	18 (38%)	0.002
Informal teaching of TUS from seniors or peers	70 (75%)	19 (40%)	<0.001
Use of intra-pleural fibrinolytic and DNase for pleural infection	25 (30%)	21 (45%)	0.028
Repeated thoracentesis as the preferred management for patients with recurrent MPE	34 (42%)	9 (19%)	0.010
Clamping chest drain prior to removal in pneumothorax	31 (66%)	37 (45%)	0.023
Consultation to thoracic surgery for surgical pleurodesis in prevention of recurrent pneumothorax	34 (42%)	36 (77%)	<0.001
Use of autologous blood patch for persistent air leak	11 (13%)	38 (60%)	<0.001

TUS Transthoracic ultrasonography, MPE Malignant pleural effusion

All respiratory physicians had undertaken certain forms of training or learning in transthoracic ultrasonography (TUS) through workshops, courses, overseas attachment, self-learning or learning from peers and seniors. On the contrary, twenty-two non-respiratory clinicians (27%) had no training or learning (including self-learning) on TUS utilization. Among respiratory physicians, 18 of 47 (38%) respiratory physicians (11 higher physician trainees and 7 respiratory specialists) had completed formal workshops, overseas courses or attachment on TUS, compared to 14% of non-respiratory physicians (x^2^ =10.615, df=1, *p*=0.002). Most respondents (98%) had access to portable ultrasound devices in their hospitals. All respiratory physicians used ultrasound to aid diagnosis of pleural diseases and guide pleural procedures in their practice, while only 86% of non-respiratory physicians performed thoracic ultrasonography in their daily clinical practice.

Regarding the scope of practice, 96% and 60% of non-respiratory physicians performed thoracentesis and closed needle pleural biopsy respectively. Eighty two percent of them would consider the utilization of TUS to guide the procedures. On the contrary, all physicians working in respiratory stream would use TUS to guide thoracentesis and/or pleural biopsy.

Fifty-eight percent of the respondents reported that trainees performed the majority of chest tube insertion procedures in their hospitals, with the remainder performed by interns, medical specialists, radiologists, thoracic surgeons or emergency physicians. However, only 26% of the basic and higher physician trainees had received formal thoracic ultrasonography training program and 70% of them described that their TUS training was inadequate. Teaching by seniors and/or peers during clinical duties was the predominant mode of learning on ultrasonography among the local clinicians (40% of respiratory physicians and 85% of non-respiratory physicians, x^2^ =28.205, df=1, *p*<0.001). The commonest reasons for referring patients to radiologists for ultrasound-guided pleural procedures were better equipment (50 %) or expertise (40%) in ultrasonography, and lack of manpower/space in medical wards (18%). Only 5% of all respondents considered the waiting time to procedures could be shorter under radiology departments.

b) *Investigations of exudative pleural effusion*

For cases with exudative pleural effusion of undetermined cause after first thoracentesis, up to 98% of the respondents would consider non-surgical pleural procedures as the next investigation. Closed blind pleural biopsy was the most frequently performed procedure (55%), followed by pleuroscopy (medical thoracoscopy) under conscious sedation (23%), and image-guided pleural biopsy (12%). Although flexi-rigid pleuroscopy service was available in all the participating hospitals, 39 (30%) respondents were unaware of the availability of this interventional service and all were non-respiratory clinicians. Most respiratory physicians (77%) had experience in performing pleuroscopy. During pleuroscopy, parietal pleural biopsy was the most commonly performed procedure (97%), followed by talc pleurodesis (57%). Twenty-six respondents (20%) reported the availability of rigid pleuroscopy under local anaesthesia in their hospitals, while 42 (33%) reported unavailability and 61 (47%) had no idea whether rigid pleuroscopy was available or not.

iii) *Management of pleural infection*

Among respiratory doctors (*n*=47), twenty-one (45%) would consider or had prescribed intra-pleural tissue plasminogen activator (tPA) and DNase therapy for managing pleural infections. In comparison, majority of non-respiratory respondents (*n*=57, 70%) were unaware of the combination intra-pleural therapy as a potential management option for poorly drained pleural infection (x^2^=4.8, df=1, *p*=0.028). Among the respiratory clinicians who did not consider the use of this combination intra-pleural therapy (*n*=26), the main reasons were unavailability of the medications in pharmacy for immediate use (85%) and concerns on the high drug cost (62%). Significantly more clinicians working in hospitals with on-site thoracic surgeons would consider to use intra-pleural therapy for improving drainage in pleural infection (Table 3), than those working in hospitals without thoracic surgery (x^2^= 6.75, df 1, *p*=0.009), indicating that availability of on-site thoracic surgery was not a major factor affecting the prescription of combination intra-pleural therapy.

**Table 3:** The response on use of intra-pleural fibrinolytic and DNase for pleural infection according to the hospital setting with or without on-site thoracic surgery

	Total number of survey participants (*n*=129)	
	Hospitals with on-site thoracic surgical unit (*n*=43)	Hospitals without on-site thoracic surgical unit (*n*=86)
Use of intra-pleural fibrinolytic and DNase for pleural infection^b^	Yes: 22	Yes: 24
	No: 21	No: 62
	Reasons of not using intra-pleural fibrinolytic and DNase for pleural infection^a^	
	*N*=21	*N*=62
• Medications not readily available	11 (52%)	22 (35%)
• More confidence in thoracic surgical approach	1 (5%)	3 (5%)
• Concerns on side effects	2 (10%)	3 (5%)
• Concerns on drug cost not covered by public funding	7 (33%)	13 (21%)
• Not aware of this treatment	4 (19%)	21 (34%)
• Others/not specified	4 (19%)	10 (16%)

^a^Respondents could choose one or more reasons

^b^ x^2^= 6.75, df 1, *p*=0.009 by chi-square test

Despite lacking benefits in clinical outcomes [11, 12], intra-pleural urokinase monotherapy was still commonly prescribed (among 64% of respiratory doctors and 40% of non-respiratory doctors) for pleural infection, given its lower cost and wider availability compared to tPA and DNase.

iv) *Management of malignant pleural effusion*

**Fig. 1:**
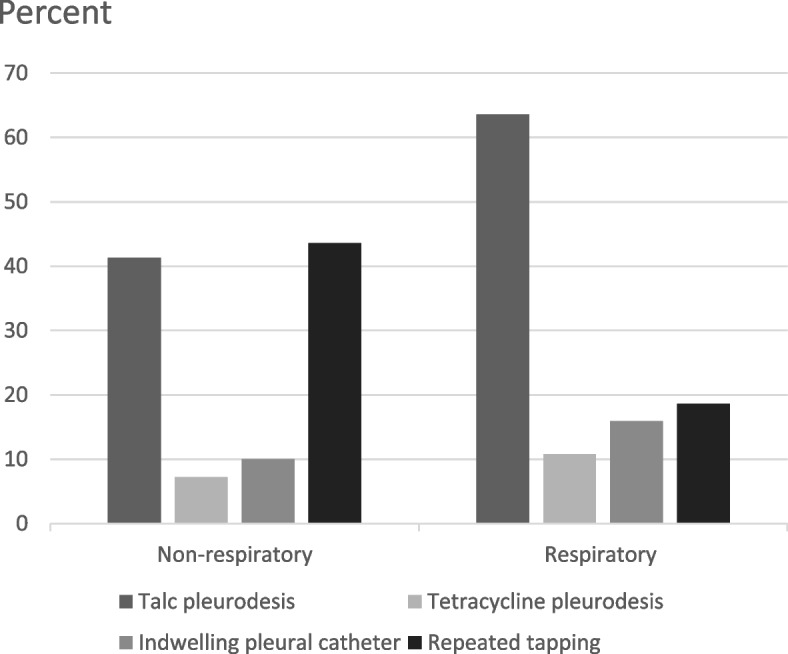
Approach to recurrent malignant pleural effusion among respiratory and non-respiratory clinicians

In the survey, 120 (93%) respondents had to manage patients with recurrent malignant pleural effusions (MPE) in their clinical practice. With such patients, repeated thoracentesis was the preferred management option among 42% of non-respiratory clinicians, as compared to 19% of respiratory clinicians (x^2^ =6.694, df=1, *p*=0.01). Majority of the respiratory clinicians (88%) preferred definitive fluid control measures with chemical pleurodesis (72%) or indwelling pleural catheter (IPC) insertion (16%) for patients with recurrent MPE (Figure 1). The survey revealed that IPC service was available in all hospitals and 66% of the respiratory respondents were able to perform the procedure. Twelve (15%) non-respiratory clinicians was not aware of IPC as an available treatment option.

e) *Management of Pneumothorax*

Among the respondents, 122 (95%) managed pneumothoraces as part of their clinical practice. At the time of the survey, one-third of the respondents (28% non-respiratory, 36% respiratory clinicians) had performed needle aspiration as an in-patient management for pneumothorax. Among those who had performed needle aspiration, 89%, 50% and 50% would consider the procedure for primary spontaneous pneumothorax (PSP), secondary spontaneous pneumothorax (SSP) and iatrogenic pneumothorax respectively. There was no significant difference regarding the use of needle aspiration for different types of pneumothorax among respiratory versus non-respiratory clinicians.

The preference on the size of chest drain for drainage of pneumothorax varied. Fifty eight (70%) non-respiratory clinicians opted for chest drain of less than 18 French for drainage of PSP, while 27 (57%) respiratory clinicians preferred chest drain of less than 18 French for PSP (x^2^ =0.535, df =1, *p*>0.05). For SSP, significantly more respiratory clinicians (59%) preferred chest drain of 20 French or more, as compared to non-respiratory clinicians (38%, x^2^ =9.276 df=1, *p*=0.003). Twelve (9%) of the respondents would apply suction immediately after chest drain was inserted for large pneumothorax, and majority (*n*=10) were non-respiratory clinicians.

Digital chest drain system was reported to be available for monitoring air leak in the institutions of 55% of the respondents. Forty-four (34%) non-respiratory clinicians were unaware of the device. Sixty-six percent of respiratory clinicians, as compared to 45% non-respiratory clinicians (x^2^ =5.169, df=1, *p*=0.023) would clamp the chest drain to check out for small or intermittent air leak and any pneumothorax recurrence, prior to chest drain removal.

Pleurodesis, either by surgical means or chemical instillation via the chest tube at bedside, is the mainstay of management for prevention of pneumothorax recurrence. 77% of respiratory physicians would consult thoracic surgeons for surgical pleurodesis or other surgical techniques for recurrence prevention. In contrast, only 42% of non-respiratory physicians would consult thoracic surgery (x^2^ =13.454, df=1, *p*<0.001). For those not seeking surgical consultation, the most common reasons were patients deemed not fit for surgery under general anaesthesia, patients’ refusal for surgery and perceived comparable efficacies between bedside chest tube pleurodesis and surgical pleurodesis . The availability of on-site thoracic surgical support did not significantly affect the proportion of clinicians who opt to refer patients for surgery for prevention of pneumothorax recurrence (x^2^= 1.57, df 1, *p*=0.211) (Table 4).

**Table 4:** The response on consultation to thoracic surgery for prevention of recurrent pneumothorax according to the hospital setting with or without on-site thoracic surgery

	Total number of survey participants (*n*=129)	
	Hospitals with on-site thoracic surgical unit (*n*=43)	Hospitals without on-site thoracic surgical unit (*n*=86)
Consultation to thoracic surgery for pleurodesis as recurrence prevention for pneumothorax^b^	Yes: 27 No: 16	Yes: 44 No: 42
	Reasons of not consulting thoracic surgeon on pleurodesis as recurrence prevention for pneumothorax^a^	
	*N*=16	*N*=42
• Patients were not fit for surgery	16 (100%)	35 (83%)
• Patients refused surgery	12 (75%)	13 (31%)
• No thoracic surgeon on site	2 (13%)	24 (57%)
• Efficacy of chemical pleurodesis via chest drain was comparable with surgical approach	4 (25%)	13 (31%)
• Others/not specified	5 (31%)	8 (19%)

^a^Respondents could choose one or more reasons

^b^x^2^= 1.57, df 1, *p*=0.211 by chi-square test

Autologous blood patch via chest drain was considered as an option for ameliorating persistent air leak in pneumothorax by 13% and 60% of non-respiratory and respiratory clinicians respectively (x^2^ =28.660, df=1, *p*<0.001). Most of the non-respiratory physicians did not consider the procedure, since they had not come across the procedure before (33%), or had concerns about complications (61%) such as pleural infection and clogging of chest drains. Amongst the respiratory physicians, seventy-four percent cited the limited efficacy of blood patch as their reason for not considering this treatment. Twenty-three (49%) respiratory clinicians had implanted endo-bronchial valves (EBV) for managing persistent air leaks in pneumothorax. Thirty-one (38%) of non-respiratory respondents did not know that EBV could be an option for managing persistent air leak.

## Discussion

The present survey has revealed significant variations in the approach and management of common pleural diseases among respiratory and non-respiratory clinicians in Hong Kong. The findings highlight several reasons that may contribute to the variations, including insufficiency in provision of formal training, transfer of new knowledge, and knowledge utilization in pleural medicine, which is a rapidly developing field in internal medicine. The heterogeneity in clinical care provision can potentially jeopardize the quality and safety of care for the patients, and inequity in access to standard healthcare.

Knowledge dissemination, monitoring utilization and evaluation of the effects after knowledge application are paramount in successful implementation of evidence in improving clinical management [13]. All too often implementation was unsuccessful or not sustained due to various barriers in the implementation process [14]. Pleural medicine, an example of a rapidly evolving field in internal medicine, deserves special attention as it is a prevalent medical problem, with paradigm-changing evidence and advances emerged in the recent decade in both diagnostic and therapeutic aspects within the field [6]. Our study has highlighted that insufficient structured training, knowledge transfer from the specialists to non-specialists, lack of timely resource allocation to service enhancement are the possible factors prohibiting the uptake of advances into the local practice of pleural medicine in Hong Kong. Availability of hospital medical technology is less important as a barrier to implementation in a high-income city like Hong Kong, as reflected by the wide availability of portable ultrasound devices and pleuroscopy. However, the lack of awareness and training in these technologies can lead to a delay in referral to specialists, undermined the role of these technologies in improving patients’ care, and ineffective utilization of available resources.

Shortage in healthcare professionals relative to the growth in healthcare demand has been a prevailing problem in many developed countries. The healthcare system in Hong Kong is characterised by high efficiency and low doctor-to-resident ratio (1.9 doctors per 1000 residents in 2019), and the specialists-to-resident ratio is even lower. The general internists have to manage many common medical conditions, at least in the initial phase of the diseases. Many fields within internal medicine are advancing rapidly, and it could be difficult for internists to stay upfront in every aspects of knowledge. However, the maintenance of updated knowledge and skills among clinicians in general approach to common medical diseases is considered an essential dimension to quality healthcare [15]. The niche of respiratory specialists with special interest in pleural diseases should form a leading group in provision of training to non-specialists, while the hospital administrators and policy makers should provide a facilitating environment and resources for such knowledge transfer to take place.

Transthoracic ultrasonography (TUS) has consistently been shown to be more accurate than physical examination in the selection of pleural puncture site [16], thus is effective in reducing the risks of procedure related complications [17–19]. International guidelines recommend all pleural procedures (for fluid) should be performed under TUS guidance [20]. Formal TUS training curriculum, training standards and credentialing have been in place in many countries, including the United Kingdom, Europe and the Australasia [21–24]. Clear training structure in clinician-based ultrasonography is lacking in many Asian countries including Hong Kong. In the survey, almost all of the non-respiratory clinicians (96%) had engaged in performing bedside pleural procedures, which reflects the ubiquity of service demand. The survey response has clearly highlighted the insufficiency of structured training in TUS for frontline clinicians in Hong Kong. Reliance on learning from peers and informal teaching by seniors would likely lead to inconsistency in level of skills, and confidence in image acquisition and interpretation. Apart from suboptimal use of resources, patients’ safety could be compromised with possible medico-legal implications following procedure related complications.

Pleuroscopy, or medical thorascoscopy, has been recommended as the investigation of choice for exudative pleural effusion of unknown cause [25], and the service has been established in many hospitals in Hong Kong since last decade [26]. Notably, as reviewed in the survey, a significant proportion of clinicians were unaware of the indication and availability of this diagnostic modality [27]. Inadequate awareness of the procedure and failure to engage expertise for expedited investigations would possibly lengthen the time required to diagnose and manage the conditions, adversely affect the management outcomes.

Pleural infection is increasingly prevalent, and can be associated with 3-month mortality as high as 50% amongst the elderly [28, 29]. Intra-pleural administration of the combination of tPA and DNase (the MIST-2 regimen) has been shown to be effective in improving drainage of infected pleural collection via chest tube and reducing surgical referrals for decortication [11]. However, in our study, under-awareness of the treatment options, limitations in medication access, and concerns on high drug cost are the barriers that hamper the potential of the medications in improving the patient’s outcomes. Such barriers in utilization of the medication affect particularly the outcome of the elderlies whose risk-benefit ratio for major surgery is often not favourable, and for whom less-invasive management options are often more preferred. Medical thoracoscopy has been supported in recent data as an alternative or in combination with intra-pleural fibrinolytic, for patients with pleural infections who are unfit for surgery, with promising effects in improving pleural drainage and shortening of hospital stay [30, 31]. Expertise in performing the procedure and further researches on the best timing and selection of subjects who will attain benefits from this approach is highly desirable.

Recurrent malignant pleural effusion (MPE) causes significant symptom burden to the patients, impairs quality of life, leads to repeated hospitalization and increases healthcare cost. Indwelling pleural catheter has evolved to become the first line management approach to definitive control for MPE in many developed regions of the world [32]. IPC has offered an ambulatory option for MPE drainage at home, allowing early discharge from hospital and empowering self-care [6]. Our survey reveals that IPC was available in all the participating hospitals, and yet it was not the most prevailing management approach for MPE among survey participants. Multiple factors could have contributed to the under-utilization of IPC, including the unawareness or unfamiliarity with IPC, the lack of readily available clean ward space for IPC insertion and insufficient community and outpatient support for IPC care and ambulatory drainage. The cost of drainage bottles and aftercare also prohibits the access to IPC for many patients with advanced cancers.

Almost all medical clinicians in the survey manage pneumothorax. In the survey, significant heterogeneity exists in many aspects of management of pneumothorax. Needle aspiration was recommended in the British Thoracic Society guideline as the first line management for symptomatic primary spontaneous pneumothorax (>2cm) or secondary spontaneous pneumothorax of modest size (1-2cm) [33]. However, in our survey, needle aspiration was not a popular approach adopted by clinicians in Hong Kong. One possible reason against its widespread use in medical wards could be the low success rate (15% in a previous study) [34]. Moreover, delaying the drain insertion can possibly slow down the turnover of beds in local public hospitals [35]. While the published guidelines are mostly based on research studies from the European and American healthcare systems, the barriers to guideline implementation need to be analysed so that more specific strategies tailored to the local setting can be developed and evaluated.

In this survey, quite a lot of clinicians were not aware of the management options such as digital drainage systems, autologous blood patch pleurodesis and implantation of endobronchial valves for persistent air leaks, which may be of benefits in the management of difficult cases of pneumothoraces [36]. As such, apart from the need for education and relevant training, establishment of specialized pleural units under respiratory physicians can potentially improve the management quality of patients with difficult pleural diseases [37, 38]. Specialised pleural units should also take the lead in conducting research studies incorporating the perspectives of local healthcare setting and demand, which more accurately inform the management standard for the benefits of the patients.

The present survey study has several limitations. The survey targeted doctors working in local public hospitals, and therefore the results cannot be generalized to other specialties or clinicians of the private sector. The survey adopted a non-random non-probability sampling, and it is plausible that the participants agreeable to the survey could be more attentive to knowledge about pleural diseases than others, leading to possible selection bias. Although the participants were instructed to answer the questions independently and return the questionnaire as soon as possible, their response might be influenced by external factors. Notwithstanding, the questionnaire responses were collected anonymously by the centre coordinators who were not involved in the process of data entry and analyses, and research personnel responsible for data management were delinked from the survey participants. As such, analytical bias is avoided.

The survey has shed light on the knowledge-action gaps in the management of common pleural disease in healthcare system of Hong Kong. To improve the quality of care for patients with pleural diseases, strategic re-organization in care delivery with establishment of specialized pleural units for management of difficult cases, provision of structural training to clinicians, timely introduction of advances in pleural medicine, and dedicated resources to support clinical care and research tailored to the local healthcare setting are important.

## Supplementary Information

**Additional file 1.**

## Data Availability

The datasets used and/or analysed during the current study are available from the corresponding author on reasonable request

## Abbreviations

TUS: Transthoracic ultrasonography
tPA: Alteplase
MPE: Malignant pleural effusion
PSP: Primary spontaneous pneumothorax
SSP: Secondary spontaneous pneumothorax
IPC: Indwelling pleural catheter
EBV: Endobronchial valve

## References

[1] Tian P, Qiu R, Wang M (2021). Prevalence, Causes, and Health Care Burden of Pleural Effusions Among Hospitalized Adults in China. JAMA Netw Open..

[2] Mummadi SR, Stoller JK, Lopez R, Kailasam K, Gillespie CT, Hahn PY (2021). Epidemiology of adult pleural disease in the United States. Chest..

[3] Psallidas I, Kalomenidis I, Porcel JM, Robinson BW, Stathopoulos GT (2016). Malignant pleural effusion: from bench to bedside. Eur Respir Rev..

[4] Kanellakis NI, Wrightson JM, Gerry S (2022). The bacteriology of pleural infection (TORPIDS): an exploratory metagenomics analysis through next generation sequencing. Lancet Microbe..

[5] Thomas R, Rahman NM, Maskell NA, Lee YCG (2020). Pleural effusions and pneumothorax: Beyond simple plumbing: Expert opinions on knowledge gaps and essential next steps. Respirology (Carlton, Vic.).

[6] Lui MMS, Lee YCG (2020). Twenty-five years of respirology: advances in pleural disease. Respirology (Carlton, Vic.).

[7] Kelly AM, Clooney M (2008). Deviation from published guidelines in the management of primary spontaneous pneumothorax in Australia. Int Med J.

[8] Reuter S, Lindgaard D, Laursen C, Fischer BM, Clementsen PF, Bodtger U (2019). Computed tomography of the chest in unilateral pleural effusions: outcome of the British Thoracic Society guideline. J Thorac Dis..

[9] Medlinskiene K, Tomlinson J, Marques I, Richardson S, Stirling K, Petty D (2021). Barriers and facilitators to the uptake of new medicines into clinical practice: a systematic review. BMC Health Serv Res.

[10] SurveyMonkey®. https://www.surveymonkey.com/mp/sample-size-calculator/. Accessed Sept 2022.

[11] Rahman NM, Maskell NA, West A (2011). Intrapleural use of tissue plasminogen activator and DNase in pleural infection. New Engl J Med.

[12] Maskell NA, Davies CW, Nunn AJ (2005). U.K. Controlled trial of intrapleural streptokinase for pleural infection. New Engl J Med.

[13] Zhao J, Bai W, Zhang Q (2022). Evidence-based practice implementation in healthcare in China: a living scoping review. Lancet Regional Health Western Pacific..

[14] Geerligs L, Rankin NM, Shepherd HL, Butow P (2018). Hospital-based interventions: a systematic review of staff-reported barriers and facilitators to implementation processes. Implement Sci.

[15] Mosadeghrad AM (2014). Factors influencing healthcare service quality. Int J Health Policy Manage.

[16] Diacon AH, Brutsche MH, Solèr M (2003). Accuracy of pleural puncture sites: a prospective comparison of clinical examination with ultrasound. Chest..

[17] Duncan DR, Morgenthaler TI, Ryu JH, Daniels CE (2009). Reducing iatrogenic risk in thoracentesis: establishing best practice via experiential training in a zero-risk environment. Chest..

[18] Mercaldi CJ, Lanes SF (2013). Ultrasound guidance decreases complications and improves the cost of care among patients undergoing thoracentesis and paracentesis. Chest..

[19] Millington SJ, Koenig S (2018). Better with ultrasound: pleural procedures in critically ill patients. Chest..

[20] Havelock T, Teoh R, Laws D, Gleeson F (2010). Pleural procedures and thoracic ultrasound: British thoracic society pleural disease guideline 2010. Thorax..

[21] Stanton AE, Edey A, Evison M, et al. British Thoracic Society Training Standards for Thoracic Ultrasound (TUS). BMJ Open Respir Res.e. 2020;7(1). 10.1136/bmjresp-2019-000552 PMC7245450 32430401

[22] Williamson JP, Twaddell SH, Lee YC (2017). Thoracic ultrasound recognition of competence: a position paper of the Thoracic Society of Australia and New Zealand. Respirology (Carlton, Vic.).

[23] Laursen CB, Clive A, Hallifax R, et al. European Respiratory Society statement on thoracic ultrasound. Eur Respir J. 2021;57(3). 10.1183/13993003.01519-2020 33033148

[24] McCracken DJ, Bedawi EO, Stevenson M, Cullen KM, Stanton AE, Rahman NM. Thoracic ultrasound competence for ultrasound guided pleural procedures: The creation and validation of an assessment tool for use in the certification of basic thoracic ultrasound competence. J Clin Ultrasound. 2022. 10.1002/jcu.23137 35034353

[25] Lee P, Colt HG (2007). State of the art: pleuroscopy. J Thorac Oncol.

[26] Law WL, Chan J, Lee S (2008). Pleuroscopy: our initial experience in Hong Kong. Hong Kong Med J..

[27] Yap KH, Phillips MJ, Lee YC (2014). Medical thoracoscopy: rigid thoracoscopy or flexi-rigid pleuroscopy?. Curr Opin Pulm Med..

[28] Addala DN, Bedawi EO, Rahman NM (2021). Parapneumonic Effusion and Empyema. Clin Chest Med..

[29] Corcoran JP, Psallidas I, Gerry S, et al. Prospective validation of the RAPID clinical risk prediction score in adult patients with pleural infection: the PILOT study. Eur Respir J. 2020;56(5). 10.1183/13993003.00130-2020 32675200

[30] Mondoni M, Saderi L, Trogu F (2021). Medical thoracoscopy treatment for pleural infections: a systematic review and meta-analysis. BMC Pulm Med..

[31] Kheir F, Thakore S, Mehta H (2020). Intrapleural fibrinolytic therapy versus early medical thoracoscopy for treatment of pleural infection. randomized controlled clinical trial. Ann Am Thorac Soc..

[32] Walker S, Mercer R, Maskell N, Rahman NM (2020). Malignant pleural effusion management: keeping the flood gates shut. Lancet Respir Med..

[33] MacDuff A, Arnold A, Harvey J (2010). Management of spontaneous pneumothorax: British Thoracic Society Pleural Disease Guideline 2010. Thorax..

[34] Chan JW, Ko FW, Ng CK (2009). Management of patients admitted with pneumothorax: a multi-centre study of the practice and outcomes in Hong Kong. Hong Kong Med J..

[35] Tschopp JM, Bintcliffe O, Astoul P (2015). ERS task force statement: diagnosis and treatment of primary spontaneous pneumothorax. Eur Respir J..

[36] Dugan KC, Laxmanan B, Murgu S, Hogarth DK (2017). Management of Persistent Air Leaks. Chest..

[37] Evison M, Blyth KG, Bhatnagar R (2018). Providing safe and effective pleural medicine services in the UK: an aspirational statement from UK pleural physicians. BMJ Open Respir Res..

[38] Hooper CE, Lee YC, Maskell NA (2010). Setting up a specialist pleural disease service. Respirology (Carlton, Vic.).

