# Supplementary material for: Implementation of evidence on management of pleural diseases: insights from a territory-wide survey of clinicians in Hong Kong
Source: BMC Pulm Med. 2022 Oct 24;22:386. doi: 10.1186/s12890-022-02196-4 (PMC9590185; doi:10.1186/s12890-022-02196-4)
Supplement: Supplementary file 1 — Additional file 1. [file 12890_2022_2196_MOESM1_ESM.docx]

**A questionnaire survey on the practice of pleural medicine in Hong Kong**

It would take about 15 – 20 minutes in completing this survey. The survey response will be anonymous. We highly appreciate your help in completing this survey.

**Please circle *ONE* best-fit answer, unless otherwise specified.**

Part A Individual Profile

1. **Your experience: years after graduation:**
2. < 5 years;
3. 5-10 years;
4. >10-20 years;
5. >20 years
6. **What is your specialty:**
7. Medicine (Other than Respiratory);
8. Respiratory Medicine ;
9. thoracic surgery;
10. Others____________________________
11. **Your job description:**
12. Basic physician trainee
13. Higher physician trainee;
14. Resident Specialist;
15. Supervisory role: AC/SMO/Consultant (Please circle)
16. Others: _____________________ (please write)
17. **Name of your institution:** _________________________________________________________

Part B Pleural procedures

1. **Do you perform thoracic USG in your practice?**
2. No;
3. Yes and under supervision;
4. Yes and independently
5. **Which of the below best describes your training in Thoracic USG?**
6. No formal training
7. self-learning
8. received teaching and supervision on thoracic USG from peers or seniors
9. completed a workshop of ≤ 1 day duration
10. completed a training course/fellowship/overseas attachment of > 1 day
11. **What sort of procedures you would perform with thoracic USG? (can circle more than one)**
12. I do not perform thoracic USG
13. inspection only;
14. pleural tapping;
15. pleural biopsy
16. FNAC lung biopsy;
17. Trucut biopsy;
18. intercostal drainage for pleural effusions;
19. intercostal drainage for pneumothorax;
20. before pleuroscopy
21. b-i of the above
22. **How often do you use thoracic USG in the procedures during pleural tapping?**
23. not at all, I perform percussion only;
24. < 33%;
25. 33- 66%;
26. 66% - 100%
27. I do not perform pleural tapping
28. **Do you perform closed pleural biopsy?**
29. Yes;
30. No
31. **Which one of the following best describes your next step of investigations for exudative effusions of unknown origin after pleural tapping?**
32. repeat tapping;
33. closed pleural biopsy;
34. image-guided (USG or CT) pleural biopsy;
35. pleuroscopy;
36. VATS;
37. I do not treat pleural effusions
38. **Do you perform pleuroscopy under local anaesthesia/conscious sedation?**
39. No (go to Q.13) ;
40. Yes and under supervision;
41. Yes and independently
42. **What sort of procedures you would perform during pleuroscopy? (can circle more than one)**
43. parietal pleural biopsy;
44. visceral pleural biopsy;
45. lung biopsy;
46. talc pleurodesis;
47. Others: ___________________ (please write)

Part C Management of Pneumothorax (PNX)

1. **Do you perform pleural aspiration for PNX?**
2. No, I seldom perform pleural aspiration for any sort of PNX (go to Q15);
3. Yes
4. **Which type(s) of PNX would you perform pleural aspiration? (can circle more than one)**
5. Primary spontaneous pneumothorax (PSP);
6. Secondary spontaneous pneumothorax (SSP);
7. Iatrogenic PNX
8. **For large PNX, do you apply suction immediately after intercostal drainage?**
9. Yes;
10. No;
11. I seldom issue the order of ‘suction’
12. **Do you ever clamp chest drains in cases with pneumothorax? (can circle more than one)**
13. No
14. Yes, during change of tubing
15. Yes, before removal of chest drain
16. Yes, during chemical pleurodesis
17. Yes, with other reasons __________________________________________ (please write)
18. **For PSP, what drain size do you prefer for drainage? (please circle one answer)**
19. small bore (≤ 12F);
20. 14 – 18F;
21. 20-24F;
22. >24F;
23. I do not put in drains
24. **For SSP, what drain size do you prefer for drainage? (please circle one answer)**
25. small bore (≤ 12F);
26. 14 – 18F;
27. 20-24F;
28. >24F;
29. I do not put in drains
30. **Which agent is your preferred choice for chemical pleurodesis in PNX? (please circle one answer)**
31. tetracycline derivatives;
32. talc;
33. others: ___________________________________________ (please write)
34. I do not order pleurodesis
35. **Do you consult thoracic surgeons for consideration of surgical pleurodesis before bedside chemical pleurodesis?**
36. No;
37. Yes, less than 50%;
38. Yes, more than 50%
39. **What are the reasons of not consulting surgeons for surgical pleurodesis? (can circle more than one)**
40. I do not make such consultations;
41. patients are usually considered not fit for surgery;
42. patients refuse surgery;
43. surgeons not available on-site;
44. Efficacy of bedside chemical pleurodesis is not much different from surgical pleurodesis
45. others _________________________________________ (please write)
46. **Do you use autologous blood patch via chest drain for persistent airleak (PAL)/pleurodesis?**
47. No; (go to question 23)
48. Yes (go to question 24)
49. **I do not consider autologous blood patch as an option in management of PAL/pleurodesis, because: (Can circle more than one)**
50. Limited efficacy;
51. A messy procedure;
52. Risk of side effects e.g. pleural infection, clogged chest tube;
53. I am not trained in doing this procedure
54. Others: __________________________________________________________________
55. **Do you implant endobronchial valves for persistent air leakage?**
56. No;
57. Yes and under supervision;
58. Yes and independently

Part D Management of Pleural Effusions

1. **Do you use intra-pleural Alteplase (tPA) and DNase in managing pleural infection?**
2. Yes; (go to question 27)
3. No (go to question 26)
4. **I do not use/seldom use IP tPA and DNase in optimizing drainage in pleural infection, because: (can circle more than one)**
5. It is expensive, patient may not be willing to pay as self-financed item;
6. It is not supported by public funding/hospital budgets;
7. The intra-pleural therapy carries side effects;
8. The drug is not readily available in the pharmacy of my institution;
9. I am more confident in surgical decortication under general anesthesia;
10. I am not aware of this treatment option
11. Others:______________________________________________________
12. **Do you use Urokinase in managing pleural infection?**
13. Yes;
14. No
15. **Do you insert Indwelling Pleural Catheters (IPC)?**
16. No;
17. Yes and under supervision;
18. Yes and independently
19. **Please rank the following options that best describe your approach in managing recurrent malignant effusions without trapped lung. (order 1,2,3,4…)**

____ Talc pleurodesis;

____ Tetracycline pleurodesis;

____ IPC;

____repeated tapping;

____ others: _____________________________________ (please write)

1. **Would you consider chemical pleurodesis or IPC upon the diagnosis of malignant pleural effusions before initiation of anti-cancer therapy?**
2. Yes, for most cases;
3. Yes, only in selected cases who stand high risk of pleural effusion re-accumulation;
4. No
5. I do not manage malignant pleural effusion

Part E Information on your institution/practice

31-46. **In your institution/place, is there the availability of: (please tick the answer on right side)**

1. Portable bedside thoracic USG machine? __ Yes; __ No; __don’t know
2. Flexi-rigid pleuroscopy service? __ Yes; __ No; __don’t know
3. Rigid LA pleuroscopy service? __ Yes; __ No; __don’t know
4. Credentialing mechanism for performing pleuroscopy? __ Yes; __ No; __don’t know
5. Credentialing mechanism for performing USG __ Yes; __ No; __don’t know
6. talc powder? __ Yes; __ No; __don’t know
7. IPC implantation service? __ Yes; __ No; __don’t know
8. Alteplase / DNAase for empyema? __ Yes; __ No; __don’t know
9. Portable digital suction machines for chest drains? __ Yes; __ No; __don’t know
10. Endobronchial valve implantation for PNX? __ Yes; __ No; __don’t know
11. Seldinger small-bore catheter insertion service by physicians? __ Yes; __ No; __don’t know
12. On-site thoracic surgeons? __ Yes; __ No; __don’t know
13. **Estimated percentage of physician-performed thoracic USG +/- pleural procedures in medical wards in the past year was:**
14. not at all (cases were referred to radiologists or surgeons for the procedure);
15. < 33%;
16. 33-66%;
17. >66%
18. **Why are cases being referred to radiologists for USG guided pleural procedures? (can circle more than one)**
19. better equipment (e.g. special drains or better USG machines)
20. better expertise and skills
21. referred by non-pulmonologists
22. FNAC or trucut lung biopsies
23. shorter waiting time
24. Short of manpower or space in medical wards for proper procedure
25. Others: ____________________________________________(please specify)
26. **Do you consider the training in thoracic USG adequate in Hong Kong?**
27. Yes;
28. No;
29. don’t know
30. **Who performs most chest drainage in your institutions?**
31. Interns;
32. Med Resident trainees;
33. Med Resident specialists or above;
34. AED doctors;
35. Thoracic surgeons;
36. Radiologists;
37. We don’t insert chest drains

**End of the questionnaire. Thank you**
